# Risk Factors Associated With Bleeding in Children With Cardiac Disease Receiving Extracorporeal Membrane Oxygenation: A Multi-Center Data Linkage Analysis

**DOI:** 10.3389/fcvm.2021.812881

**Published:** 2022-01-13

**Authors:** Ashish A. Ankola, David K. Bailly, Ron W. Reeder, Katherine Cashen, Heidi J. Dalton, Stephen J. Dolgner, Myke Federman, Rod Ghassemzadeh, Adam S. Himebauch, Asavari Kamerkar, Josh Koch, Joseph Kohne, Margaret Lewen, Neeraj Srivastava, Renee Willett, Peta M. A. Alexander

**Affiliations:** ^1^Department of Pediatrics, Divisions of Critical Care and Cardiology, Baylor College of Medicine, Houston, TX, United States; ^2^Department of Pediatrics, Division of Pediatric Critical Care, University of Utah, Salt Lake City, UT, United States; ^3^Department of Pediatrics, University of Utah, Salt Lake City, UT, United States; ^4^Department of Pediatrics, Children's Hospital of Michigan, Detroit, MI, United States; ^5^Department of Pediatrics and Heart and Vascular Institute, Inova Fairfax Hospital, Fall Church, VA, United States; ^6^Department of Pediatrics, Division of Cardiology, Baylor College of Medicine, Houston, TX, United States; ^7^Department of Pediatrics, Mattel Children's Hospital UCLA, Los Angeles, CA, United States; ^8^Department of Critical Care Medicine, Children's Hospital of Pittsburgh, Pittsburgh, PA, United States; ^9^Department of Anesthesiology and Critical Care, Children's Hospital of Philadelphia, Philadelphia, PA, United States; ^10^Department of Anesthesiology and Critical Care Medicine, Children's Hospital Los Angeles, Los Angeles, CA, United States; ^11^Department of Pediatrics, Phoenix Children's Hospital, Phoenix, AZ, United States; ^12^Department of Pediatrics, University of Michigan, Ann Arbor, MI, United States; ^13^Department of Pediatrics, Children's National Hospital, Washington, DC, United States; ^14^Department of Cardiology, Boston Children's Hospital, Boston, MA, United States; ^15^Department of Pediatrics and Harvard Medical School, Boston, MA, United States

**Keywords:** extracorporeal membrane oxygenation, bleeding, pediatric, cardiac, anticoagulation

## Abstract

**Background:** Bleeding is a common complication of extracorporeal membrane oxygenation (ECMO) for pediatric cardiac patients. We aimed to identify anticoagulation practices, cardiac diagnoses, and surgical variables associated with bleeding during pediatric cardiac ECMO by combining two established databases, the Collaborative Pediatric Critical Care Research Network (CPCCRN) Bleeding and Thrombosis in ECMO (BATE) and the Extracorporeal Life Support Organization (ELSO) Registry.

**Methods:** All children (<19 years) with a primary cardiac diagnosis managed on ECMO included in BATE from six centers were analyzed. ELSO Registry criteria for bleeding events included pulmonary or intracranial bleeding, or red blood cell transfusion >80 ml/kg on any ECMO day. Bleeding odds were assessed on ECMO Day 1 and from ECMO Day 2 onwards with multivariable logistic regression.

**Results:** There were 187 children with 114 (61%) bleeding events in the study cohort. Biventricular congenital heart disease (94/187, 50%) and cardiac medical diagnoses (75/187, 40%) were most common, and 48 (26%) patients were cannulated directly from cardiopulmonary bypass (CPB). Bleeding events were not associated with achieving pre-specified therapeutic ranges of activated clotting time (ACT) or platelet levels. In multivariable analysis, elevated INR and fibrinogen were associated with bleeding events (OR 1.1, CI 1.0–1.3, *p* = 0.02; OR 0.77, CI 0.6–0.9, *p* = 0.004). Bleeding events were also associated with clinical site (OR 4.8, CI 2.0–11.1, *p* < 0.001) and central cannulation (OR 1.75, CI 1.0–3.1, *p* = 0.05) but not with cardiac diagnosis, surgical complexity, or cannulation from CPB. Bleeding odds on ECMO day 1 were increased in patients with central cannulation (OR 2.82, 95% CI 1.15–7.08, *p* = 0.023) and those cannulated directly from CPB (OR 3.32, 95% CI 1.02–11.61, *p* = 0.047).

**Conclusions:** Bleeding events in children with cardiac diagnoses supported on ECMO were associated with central cannulation strategy and coagulopathy, but were not modulated by achieving pre-specified therapeutic ranges of monitoring assays.

## Introduction

Bleeding is a common complication in children supported on extracorporeal membrane oxygenation (ECMO) and has been associated with increased mortality (1, 2). Consensus regarding optimal anticoagulation and monitoring strategies are lacking, despite multiple retrospective and observational studies (3–9). Center level variability in choice of anticoagulant, monitoring assays and associated therapeutic targets has been documented in surveys of ECMO center directors (3, 10). The Bleeding and Thrombosis on ECMO (BATE) prospective observational study examined risk factors for bleeding and thrombosis in pediatric patients supported with ECMO. Proportions of patients with bleeding and thrombotic complications varied between the eight BATE study sites suggesting that center variation may contribute to differences in patient outcomes (1).

Children with cardiac diagnoses may be at increased risk of bleeding during systemic anticoagulation required for ECMO, due to factors including coagulopathy post cardio-pulmonary bypass (CPB), trans-thoracic cannulation, and multiple cannulation sites (1, 11, 12). Our primary objective was to investigate the relationship between the clinical team's anticoagulation goals for children on ECMO with bleeding outcomes in the higher risk cardiac population included in the BATE dataset. For this analysis, we chose to leverage the granular anticoagulation monitoring data in the BATE dataset with the robust diagnostic and procedural data reported to the Extracorporeal Life Support Organization (ELSO) Registry. As a secondary objective, we sought to identify factors associated with mortality in the study cohort.

## Methods

### Setting and Subjects

This study was a secondary analysis of data collected for the BATE study linked with data entered into the ELSO registry. The BATE study enrolled patients <19 years of age treated with ECMO at eight participating centers between December 2012 and September 2014 (1). Data at the ECMO run level were linked by each of six participating centers utilizing medical record numbers or financial identification numbers to match patient BATE ID and ELSO Run ID numbers. Patients whose indication for ECMO was cardiac or extracorporeal cardiopulmonary resuscitation (ECPR) were included in this study cohort. The study was approved with a waiver of informed consent by the Institutional Review Boards at each of the participating hospitals and the Data Coordination Center at the University of Utah.

### Clinical Data and Definitions—BATE/CPCCRN

As described in prior BATE CPCCRN studies, daily data collection tools were completed by trained bedside ECMO specialists and research coordinators via direct observation, discussion with clinicians, and review of records. Data included demographics, primary ECMO indication, type of pump, mode of ECMO, duration of ECMO, intensive care unit (ICU) and hospital length of stay, number of failed organs, cannulation to ECMO from CPB, clinical site, acute diagnosis and chronic diagnoses. Daily results of anticoagulation and hemostatic laboratory results closest to 07:00 A.M. were collected (1). Hemostatic targets were established for each patient by their medical team and recorded daily. Based on their daily lab values and set targets for each assay the day prior, patients were classified as either “high,” “low” or “at target.” Daily heparin dose (IU/kg/h) was collected, excluding heparin used to prime the circuit or for line patency.

Bleeding complications were defined as blood loss requiring any transfusion or intracranial hemorrhage in the initial dataset. For the purposes of this analysis, we evaluated risk factors associated with “clinically important” bleeding events defined as pulmonary or intracranial hemorrhage, or massive transfusion (>80 ml/kg of total blood products) on any study day (13). In order to minimize the influence of early postoperative bleeding secondary to intra-operative anticoagulation techniques on patients post-cardiotomy, and to allow enough time for routine ECMO anticoagulation management to influence bleeding, bleeding events that occurred during the first day on ECMO were excluded from the primary analysis. As a result, subjects with <24 h of ECMO were also excluded.

### Clinical Data and Definitions—ELSO

Linked data obtained from the ELSO registry for eligible BATE subjects included cardiac diagnoses and cardiac surgical procedures. Specific cardiac diagnoses were identified utilizing *International Classification of Diseases*, 9th Revision (ICD-9) diagnostic codes. Primary diagnosis ICD-9 codes were used to separate patients into the following categories: single ventricle congenital heart disease, biventricular congenital heart disease, cardiomyopathy or myocarditis, and pulmonary hypertension. Specific ICD-9 codes, their diagnoses, and categorization are shown in Supplementary Table 1. Cardiac surgical procedures were identified using Current Procedural Terminology (CPT) procedure codes, allowing for the categorization of patients as “cardiac surgical” and “cardiac medical.” Patients undergoing cardiac surgery only at the conclusion of their ECMO run were classified as “cardiac medical.” CPT codes indicating cardiac surgery (excluding cardiac procedure not otherwise specified, pericardiocentesis, extracorporeal support, drainage or incision of heart sac) were further grouped into The Society of Thoracic Surgeons-European Association for Cardio-Thoracic Surgery (STAT) Categories (14). CPT codes were also used to identify patients requiring mediastinal exploration during their ECMO run.

### Statistical Analysis

In order to assess factors associated with daily bleeding, a day-level dataset was constructed with one record per subject per ECMO day. Bleeding was assessed on a daily basis but the time of the event was not collected. In order to ensure that predictors of daily bleeding were assessed prior to the occurrence of the bleeding event, lab values from the prior calendar day were used as predictors (i.e.,: day 2 bleeding was predicted by lab values from day 1). This approach was also used in assessing the association of bleeding events with attainment of anticoagulation lab value targets (i.e.,: target set on day 1 informed lab value on day 2, which would predict bleeding on day 3). Logistic regression models were created to assess relationships with daily bleeding and selected a priori covariates with forced inclusion of clinical site and cardiac diagnosis. Generalized estimating equations with an autoregressive correlation structure of order 1 were used to account for clustering of daily events within a subject. Odds ratios (OR) were reported for meaningful changes in the predictors (i.e.,: flow rate is measured in mL/kg/min but the odds ratio is reported for an increase of 20 mL/kg/min).

A multivariable model for bleeding odds was developed using selected variables based on clinical judgement after considering their significance in modeling, the percentage of missing values for the variable, and their collinearity with other variables. Clinical site and cardiac diagnostic groups were forced into the multivariable model because site factors and specific cardiac diagnoses may affect both clinical practice and bleeding risk. Variables included were clinical site, cardiac diagnostic group, age, cannulation from CPB, central cannulation, and the following from the previous study day: lactate, activated clotting time (ACT), international normalized ration (INR), fibrinogen, platelets, ACT relative to target, and platelets relative to target. Each possible subset of these potential predictors was used to create a multivariable model and the penalized model fit was assessed using the quasilikelihood under the independence model criterion (QIC), an analog of the commonly used Akaike information criterion (AIC) that is suitable for the correlation structure of our model. The optimal model (lowest QIC) was selected as the one with the best fit to the data without overfitting; however, any model with a QIC within 2 points of the optimal model was considered statistically equivalent in terms of fit. Clinical judgement was used to select the most relevant of these near-optimal models.

Secondary analysis of factors associated with bleeding events on the 1st day of ECMO cannulation was also performed using logistic regression. Clinical site, cardiac diagnosis, and heparin bolus at initiation were forced into the model. Additional variables considered included age, central cannulation, cannulation from CPB, and the last measurement of the following prior to cannulation: lactate, INR, fibrinogen, and platelets. Near-optimal models were selected using AIC.

In hospital mortality was also summarized for this cohort. Associations with mortality were assessed with logistic regression. Multivariable models were developed analogously to prior models. Variables selected included clinical site, cardiac diagnostic group, age, cannulation from CPB, organ failure index prior to cannulation, proportion of ECMO days with bleeding and the last measurements of lactate and pH prior to cannulation.

Subject characteristics were summarized with medians and quartiles for continuous variables and with counts and percentages for categorical variables. Analyses were performed using SAS 9.4 (SAS Institute; Cary, NC). *P*-values were based on a two-sided alternative and considered significant if <0.05. Regression covariates with more than 5% missing values were set to the median value when missing and coupled with an additional covariate to indicate that the value was missing. Including these two covariates simultaneously allowed the analysis to avoid biased inference from excluding subjects with missing values; it also allowed measured values to enhance the model without the imputed value having any influence.

## Results

Patient and circuit characteristics of the 187 patients who met inclusion criteria are shown in Table 1. The majority of patients received veno-arterial (VA) ECMO, 95 (51%) patients were centrally cannulated and 46 (24%) patients were cannulated via ECPR. The majority of patients were post-operative cardiac surgical (112, 60%), most commonly STAT Category >3, and 48 (26%) patients were placed on ECMO directly from CPB. Median duration of ECMO was 4.6 days (IQR 2.8, 7.0).

**Table 1:** Subject and ECMO characteristics by occurrence of bleeding.

	**Bleeding event**		
	**No (*N* = 73)**	**Yes (*N* = 114)**	**Overall (*N* = 187)**
Clinical Site			
A	8 (11.0%)	23 (20.2%)	31 (16.6%)
B	11 (15.1%)	10 (8.8%)	21 (11.2%)
C	29 (39.7%)	22 (19.3%)	51 (27.3%)
D	8 (11.0%)	33 (28.9%)	41 (21.9%)
E	7 (9.6%)	5 (4.4%)	12 (6.4%)
F	10 (13.7%)	21 (18.4%)	31 (16.6%)
Sex			
Male	48 (65.8%)	70 (61.4%)	118 (63.1%)
Female	25 (34.2%)	44 (38.6%)	69 (36.9%)
Age Group			
Preterm neonate	2 (2.7%)	11 (9.6%)	13 (7.0%)
Full-term neonate	21 (28.8%)	47 (41.2%)	68 (36.4%)
Infant	23 (31.5%)	34 (29.8%)	57 (30.5%)
Child	18 (24.7%)	18 (15.8%)	36 (19.3%)
Adolescent	9 (12.3%)	4 (3.5%)	13 (7.0%)
E-CPR	18 (24.7%)	28 (24.6%)	46 (24.6%)
STAT category			
Cardiac medical patient	37 (50.7%)	38 (33.3%)	75 (40.1%)
Cardiac surgery not categorizable	1 (1.4%)	3 (2.6%)	4 (2.1%)
1	0 (0.0%)	2 (1.8%)	2 (1.1%)
2	11 (15.1%)	4 (3.5%)	15 (8.0%)
3	4 (5.5%)	10 (8.8%)	14 (7.5%)
4	14 (19.2%)	33 (28.9%)	47 (25.1%)
5	6 (8.2%)	24 (21.1%)	30 (16.0%)
Cardiac diagnostic group			
SV CHD	20 (27.4%)	31 (27.2%)	51 (27.3%)
BV CHD	32 (43.8%)	62 (54.4%)	94 (50.3%)
CM	9 (12.3%)	11 (9.6%)	20 (10.7%)
PH	7 (9.6%)	1 (0.9%)	8 (4.3%)
Other/Unknown	5 (6.8%)	9 (7.9%)	14 (7.5%)
Mediastinal exploration during ECMO			
No	70 (95.9%)	78 (68.4%)	148 (79.1%)
Yes	3 (4.1%)	36 (31.6%)	39 (20.9%)
Baseline organ failure index			
2	48 (65.8%)	65 (57.0%)	113 (60.4%)
3	22 (30.1%)	35 (30.7%)	57 (30.5%)
4–5	3 (4.1%)	14 (12.3%)	17 (9.1%)
Baseline blood urea nitrogen (mg/dL)	21.0 (14.0, 32.0)	15.0 (9.5, 25.5)	18.0 (10.0, 28.0)
Baseline creatinine (mg/dL)	0.7 (0.4, 1.0)	0.6 (0.4, 0.9)	0.6 (0.4, 0.9)
Baseline aspartate aminotransferase (IU/L)	131.0 (57.0, 372.0)	72.0 (37.0, 213.0)	89.0 (44.0, 288.0)
Baseline alanine aminotransferase (IU/L)	55.0 (33.5, 198.5)	34.0 (25.0, 61.0)	40.0 (27.0, 84.0)
Baseline total bilirubin (mg/dL)	2.0 (0.8, 5.9)	3.2 (1.3, 5.6)	2.7 (1.0, 5.6)
Cannulation directly from CPB			
No	62 (84.9%)	77 (67.5%)	139 (74.3%)
Yes	11 (15.1%)	37 (32.5%)	48 (25.7%)
Central cannulation			
No	47 (64.4%)	45 (39.5%)	92 (49.2%)
Yes	26 (35.6%)	69 (60.5%)	95 (50.8%)
Left atrial vent placement			
No	67 (91.8%)	102 (89.5%)	169 (90.4%)
Yes	6 (8.2%)	12 (10.5%)	18 (9.6%)
Type of pump			
Roller Head	28 (38.4%)	19 (16.7%)	47 (25.1%)
Centrifugal	45 (61.6%)	95 (83.3%)	140 (74.9%)
Average daily heparin dose (units/kg/hr)	18.7 (12.2, 25.2)	21.5 (14.8, 26.9)	20.0 (14.0, 26.5)
Mean daily ECMO flow rate (mL/kg/min)	91.4 (70.5, 118.0)	104.1 (91.2, 125.7)	100.0 (83.8, 121.9)
Duration of ECMO (days)	3.9 (2.5, 5.8)	5.3 (3.0, 7.9)	4.6 (2.8, 7.0)
ICU length of stay (days)	24.9 (13.5, 64.3)	28.1 (13.4, 48.9)	27.1 (13.4, 52.9)
Total length of stay (days)	37.0 (18.6, 77.0)	38.8 (13.9, 88.7)	38.0 (15.7, 80.9)
Vital status at hospital discharge			
Dead	31 (42.5%)	73 (64.0%)	104 (55.6%)
Alive	42 (57.5%)	41 (36.0%)	83 (44.4%)

*BV, biventricular; CHD, congenital heart disease; CM, myocarditis/cardiomyopathy; CPB, cardiopulmonary bypass; ECMO, extracorporeal membrane oxygenation; E-CPR, extracorporeal cardiopulmonary resuscitation; ICU, intensive care unit; PH, pulmonary hypertension; STAT, Society of Thoracic Surgeons-European Association of Cardiothoracic Surgery; SV, single ventricle*.

### Bleeding Events Logistic Regression Analysis

Bleeding events occurred in 114 (61%) patients. Types of bleeding events separated by cardiac medical and surgical groups are shown in Table 2. Notably, massive transfusion events occurred in 74/112 (66%) cardiac surgical and in 34/75 (45%) cardiac medical patients. Results of logistic regression modeling are shown in Supplementary Table 2. Of note, daily bleeding odds were not associated with patient age, ECPR cannulation, type of pump utilized, daily heparin dose, mediastinal exploration during the ECMO run, or increased surgical complexity by STAT categorization.

**Table 2:** Bleeding events in cardiac medical/surgical groups.

	**Surgical**	**Medical**	**Overall**
	**(*N* = 112)**	**(*N* = 75)**	**(*N* = 187)**
Pulmonary hemorrhage	9 (8%)	7 (9.3%)	16 (13.4%)
Intracranial hemorrhage	13 (11.6%)	12 (16.0%)	25 (13.4%)
Massive transfusion	74 (66.1%)	34 (45.3%)	108 (57.8%)

On multivariable logistic regression (Table 3), odds of daily bleeding were independently associated with clinical site (OR 4.8, 95% CI 2.0–11.1, *p* < 0.001). Central cannulation (OR 1.8, 95% CI 1.0–3.1, *p* = 0.050), and increased INR (OR 1.1, 95% CI 1.0–1.3, *p* = 0.028, for each 0.5 increase in INR the day prior) were associated with increased bleeding, while increased day prior fibrinogen was associated with less bleeding (OR 0.8, 95% CI 0.6–0.9, *p* = 0.004 for each 100 mg/dL increment). Notably, bleeding odds were not associated with cardiac diagnosis, ACT level compared to set target, platelet count the day prior to bleeding, or cannulation from CPB.

**Table 3:** Multivariable model of daily bleeding with near-optimal penalized fit.

	**Δ** **QIC** **=** **1.5**	
	**Odds ratio (95% CI)**	***P*-value**
Clinical site		<0.001
A	4.77 (2.04, 11.12)	
B	1.93 (0.62, 6.05)	
C	Reference	
D	1.93 (0.67, 5.57)	
E	0.89 (0.26, 3.08)	
F	3.84 (1.72, 8.56)	
Cardiac diagnostic group		0.370
BV CHD	Reference	
SV CHD	1.07 (0.64, 1.80)	
CM	0.53 (0.21, 1.37)	
PH	0.45 (0.06, 3.49)	
Other/unknown	1.67 (0.63, 4.38)	
Central Cannulation	1.75 (1.00, 3.05)	0.050
INR (0.5)	1.13 (1.01, 1.27)	0.028
Fibrinogen (100 mg/dL)	0.77 (0.64, 0.92)	0.004
Platelets (10 × 10^3^/μL)	0.98 (0.95, 1.01)	0.242
ACT compared to goal		0.191
High	1.38 (0.96, 2.00)	
Low	0.88 (0.57, 1.37)	
Not assessed, >1 day after cannulation	0.67 (0.27, 1.64)	
Not assessed, day after cannulation	1.07 (0.67, 1.72)	
Target	Reference	

*Modeling is based on the 1,046 complete records in which all potential predictors and the outcome are non-missing. ACT, activated clotting time; BV, biventricular; CHD, congenital heart disease; CM, myocarditis/cardiomyopathy; INR, international normalized ratio; PH, pulmonary hypertension; SV, single ventricle*.

### Bleeding Events on ECMO Day 1

Multivariable logistic regression of factors associated with bleeding on ECMO Day 1 (Table 4) showed that central cannulation (OR 2.8, 95% CI 1.2–7.1, *p* = 0.023) and cannulation from CPB (OR 3.3, 95% CI 1.0–11.6, *p* = 0.047) were associated with increased odds of bleeding. ECMO Day 1 bleeding was not associated with cardiac diagnosis or use of a heparin bolus at cannulation.

**Table 4:** Multivariable model of ECMO day 1 bleeding with near-optimal penalized fit.

	**Δ** **AIC** **=** **1.5**	
	**Odds ratio (95% CI)**	***P*-value**
Heparin bolus at cannulation	0.53 (0.18, 1.60)	0.257
Clinical Site		0.062
A	0.92 (0.26, 3.23)	
B	0.98 (0.21, 4.23)	
C	Reference	
D	3.53 (1.10, 11.89)	
E	0.56 (0.09, 2.91)	
F	1.75 (0.51, 6.17)	
Cardiac diagnostic group		0.671
BV CHD	Reference	
SV CHD	0.81 (0.35, 1.82)	
CM	0.91 (0.25, 3.01)	
Other/unknown	1.94 (0.48, 7.46)	
Central cannulation	2.82 (1.15, 7.08)	0.023
Cannulation directly from CPB	3.32 (1.02, 11.61)	0.047
Baseline lactate (mmol/L)	0.99 (0.92, 1.07)	0.856

*Modeling is based on the 179 complete records in which all potential predictors and the outcome are non-missing. None of the 8 subjects with pulmonary hypertension had bleeding on Day 1; these subjects are excluded from models.*

*BV, biventricular; CHD, congenital heart disease; CM, myocarditis/cardiomyopathy; CPB, cardiopulmonary bypass; SV, single ventricle*.

### Patient Outcomes

Overall mortality prior to hospital discharge was 56%. On multivariable logistic regression, odds of mortality were increased in patients with higher baseline organ failure index score (*p* = 0.009) and lower baseline pH (*p* = 0.012) prior to cannulation. Mortality was not associated with increased proportion of bleeding days during the ECMO run, cardiac diagnosis, cannulation from CPB, or increased baseline lactate (Table 5).

**Table 5:** Multivariable model of in-hospital mortality with near-optimal penalized fit.

	**Δ** **AIC** **=** **1.9**	
	**Odds ratio (95% CI)**	***P*-value**
Clinical Site		0.464
A	0.51 (0.17, 1.43)	
B	0.38 (0.12, 1.15)	
C	Reference	
D	0.63 (0.24, 1.64)	
E	0.39 (0.09, 1.58)	
F	0.86 (0.28, 2.66)	
Cardiac diagnostic group		0.606
BV CHD	Reference	
CM	1.20 (0.38, 3.97)	
Other/Unknown	1.03 (0.30, 3.73)	
PH	0.27 (0.04, 1.41)	
SV CHD	1.03 (0.49, 2.19)	
Baseline organ failure index		0.009
2	Reference	
3	2.38 (1.13, 5.14)	
4–5	5.63 (1.46, 28.66)	
Baseline arterial pH (0.1)	0.74 (0.57, 0.94)	0.012
Cannulation directly from CPB	1.44 (0.60, 3.48)	0.416
Baseline lactate (mmol/L)		
Proportion of ECMO days with bleeding (0.2)	1.13 (0.88, 1.46)	0.330

*Modeling is based on the 187 complete records in which all potential predictors and the outcome are non-missing.*

*BV, biventricular; CHD, congenital heart disease; CM, myocarditis/cardiomyopathy; CPB, cardiopulmonary bypass; ECMO, extracorporeal membrane oxygenation; PH, pulmonary hypertension; SV, single ventricle*.

## Discussion

In this multicenter registry linkage study, including granular anticoagulation, cardiac diagnostic, and procedural data, we found that clinically important bleeding events occurred in 61% of pediatric cardiac patients during ECMO. Notably, attainment of clinical team defined ACT and platelet targets, age, cardiac diagnostic group, and surgical complexity by STAT category were not associated with risk of bleeding. Central cannulation and cannulation from CPB were independently associated with increased bleeding on ECMO Day 1. Central cannulation, higher INR, lower fibrinogen, and clinical site were independently associated with increased bleeding from ECMO Day 2 onwards. In this mixed cohort of cardiac medical and surgical patients, increased bleeding events were not independently associated with mortality.

The importance of clinical site for risk of bleeding events in our analysis reflects local differences in anticoagulation management. In our cohort, attainment of pre-specified thresholds for ACT and circulating platelets was not associated with bleeding events. This has not previously been evaluated in larger, multicenter studies due to lack of granular data and the heterogeneity of institutional management of anticoagulants and hemostatic agents. Additionally, many single center studies have reported local assay targets but not evaluated bleeding outcomes according to compliance. Our findings are aligned with other studies indicating that anticoagulation assays lack correlation to heparin dosing and clinical bleeding events (5, 6, 8, 9, 15). Risk of bleeding was also not associated with platelet counts, or platelet count relationship with assay targets. Future studies of more global assays of hemostasis, such as thromboelastography, or a combination of assays coupled with the clinical scenario may be required to optimize care of this vulnerable population.

In this study, we did not find an association between risk of bleeding and underlying cardiac diagnosis or surgical complexity in pediatric cardiac patients who received ECMO. In a prior study of bleeding risk in pediatric cardiac patients receiving ECMO, the authors showed no association with CHD, but did find an increased risk of bleeding in cardiac surgical patients with higher STAT category (11). Inclusion of additional hemostatic covariates in our analyses, such as evidence of coagulopathy after CPB, and smaller sample size may explain these differences in our results. We documented a similar incidence of bleeding to previous studies and found that cumulative incidence of bleeding events was higher in patients with mortality, however there was no association between proportion of days with bleeding events and mortality. Compared to studies demonstrating increased mortality associated with bleeding events, our cohort was comprised of more cardiac medical patients than post-cardiotomy patients who may be more sensitive to deficient preload post-CPB (1, 11, 15).

An important finding from our study is that clinical factors influence bleeding risk at different times during the ECMO run. For example, cannulation to ECMO from CPB was only associated with bleeding events on the day of ECMO cannulation, suggesting that CPB effects on hemostatic derangement are limited to the immediate postoperative period. On the other hand, ongoing coagulopathy, and central cannulation continue to impact risk of bleeding from ECMO Day 2 onward in this mixed cardiac population. Coagulopathy was evidenced by increased INR and decreased fibrinogen the day prior, consistent with prior work (16). Attention to correction of coagulopathy may be important in mitigating bleeding risk in the pediatric cardiac population. The consistent association of central cannulation with increased risk of bleeding in our study and others' may reflect the impact of recent post-operative status or the predisposition of the sternal surface to bleeding, both non-modifiable aspects of patient care (11, 15).

## Limitations

This was a retrospective study and, as such, the associations between bleeding events and their risk factors do not infer causation. Additionally, as this was a multicenter cohort, anticoagulation strategies, including laboratory assay targets, and ECMO equipment varied between sites and could have impacted the primary outcome. Despite data abstraction training for the BATE dataset, the subjective nature of initially captured bleeding definitions may influence the reported rates of bleeding. Our definition of clinically important bleeding was based on clinician consensus and not on a single guideline as there is a lack of consensus in the literature. Included laboratory results were those captured closest to 7 A.M. regardless of the time of bleeding, which could further confound the relationship of assay results to identified bleeding events. We did not control for different anticoagulation assay analyzers, test solutions, or different unfractionated heparin lots across sites. We were unable to control for surgical variables such as CPB time, proportion of protamine reversal, and presence or extent of aortic suture lines as they were not collected in either dataset reliably. Lastly, we were unable to assess the relationship of bleeding to anti-Xa levels and levels of aPTT, PT and INR compared to set targets due to missing data, which may make our results less generalizable.

## Conclusions

Clinically significant bleeding events in pediatric cardiac patients cannulated to ECMO were increased with central cannulation and ongoing coagulopathy but were not associated with cardiac diagnosis nor level of ACT compared to set targets. Cannulation directly from CPB was associated with bleeding risk on the first day of ECMO, but not thereafter. This highlights the importance of coagulopathy correction in the pediatric cardiac ECMO population, regardless of underlying diagnosis. Future prospective study is needed to better understand the impact of more recent hemostatic monitoring, and the relationship to bleeding in this vulnerable population.

## Data Availability Statement

The raw data supporting the conclusions of this article will be made available by the authors, without undue reservation.

## Author Contributions

AA contributed to study design, data collection, data interpretation, drafting of the manuscript, and reviewed and revised the manuscript. DB and RR contributed to study design, data collection, data interpretation, and reviewed and revised the manuscript. PA contributed to study design, data interpretation, and reviewed and revised the manuscript. KC, MF, RG, AH, AK, JKoc, JKoh, ML, NS, and RW contributed to data collection and reviewed and revised the manuscript. SD contributed to data collection and data interpretation. HD contributed to study design and reviewed and revised the manuscript. All authors contributed to the article and approved the submitted version.

## Funding

This work was supported by the following cooperative agreements from the *Eunice Kennedy Shriver* National Institute of Child Health and Human Development, National Institutes of Health, Department of Health and Human Services: U10HD050096, U10HD049981, U10HD049983, U10HD050012, U10HD063108, U10HD063114, and U01HD049934. AH receives support not directly related to this work from the National Heart, Lung, and Blood Institute of the National Institutes of Health under Award Number K23HL153759. PA receives research funding not directly related to this work from National Institutes of Health (NIH) R13HD104432-01 Pediatric ECMO Anticoagulation Collaborative (PEACE) and her institution receives consulting funds from Novartis for PANORAMA-HF endpoint adjudication.

## Conflict of Interest

The authors declare that the research was conducted in the absence of any commercial or financial relationships that could be construed as a potential conflict of interest.

## Publisher's Note

All claims expressed in this article are solely those of the authors and do not necessarily represent those of their affiliated organizations, or those of the publisher, the editors and the reviewers. Any product that may be evaluated in this article, or claim that may be made by its manufacturer, is not guaranteed or endorsed by the publisher.
