# Supplementary material for: Risk Factors Associated With Bleeding in Children With Cardiac Disease Receiving Extracorporeal Membrane Oxygenation: A Multi-Center Data Linkage Analysis
Source: Front Cardiovasc Med. 2022 Jan 13;8:812881. doi: 10.3389/fcvm.2021.812881 (PMC8792849; doi:10.3389/fcvm.2021.812881)
Supplement: Supplementary file 1 [file Table_1.DOCX]

|  | Supplemental Table 1. Cardiac Diagnostic Group ICD-9 Codes | | | |
| --- | --- | --- | --- | --- |
|  | | |  |  |
| **Cardiac Diagnostic Group** | | ICD-9 Description | ICD-9 Code |  |
| SV CHD | | Hypoplastic Left Heart Syndrome | 746.7 |  |
|  | | Tricuspid Atresia | 746.1 |  |
|  | | Common ventricle/Double Inlet Left Ventricle | 745.3 |  |
| BV CHD | | Common Truncus | 745.0 |  |
|  | | Double Outlet Right Ventricle | 745.11 |  |
|  | | Complete Transposition of the Great Vessels | 745.10 |  |
|  | | Congenitally Corrected Transposition of the Great Arteries | 745.12 |  |
|  | | Other Transposition of Great Vessels | 745.19 |  |
|  | | Tetralogy of Fallot | 745.2 |  |
|  | | Ventricular Septal Defect | 745.3 |  |
|  | | Secundum Atrial Septal Defect | 745.5 |  |
|  | | Endocardial Cushion Defect/Atrioventricular Septal Defect | 745.60, 745.61, 745.69 |  |
|  | | Congenital Pulmonary Valve Anomaly | 746.0 |  |
|  | | Pulmonary Valve Stenosis | 746.02 |  |
|  | | Infundibular Pulmonary Stenosis | 746.83 |  |
|  | | Congenital Aortic Stenosis | 746.3 |  |
|  | | Congenital Aortic Insufficiency | 746.4 |  |
|  | | Congenital Mitral Stenosis | 746.5 |  |
|  | | Congenital Mitral Regurgitation | 746.6 |  |
|  | | Cor Triatriatum | 746.82 |  |
|  | | Coronary Artery Anomaly | 746.85 |  |
|  | | Patent Ductus Arteriosus | 747.0 |  |
|  | | Coarctation of the Aorta | 747.1 |  |
|  | | Anomaly of Aorta Unspecified | 747.20 |  |
|  | | Anomalies of Aortic Arch | 747.21 |  |
|  | | Other Anomalies of Pulmonary Arteries | 747.39 |  |
|  | | Anomaly of Great Veins | 747.40 |  |
|  | | Total Anomalous Pulmonary Venous Connection | 747.41 |  |
|  | | Partial Anomnalous Pulmonary Venous Connection | 747.42 |  |
|  | | Ebstein’s Anomaly | 746.2 |  |
|  | | Pulmonary Valve Atresia | 746.01 |  |
|  | | Other Anomalies of Bulbus Cordis/Septa | 745.8 |  |
| CM | | Diphtheritic Myocarditis | 032.82 |  |
|  | | Meningococcal Myocarditis | 036.43 |  |
|  | | Cocksackie Myocarditis | 074.23 |  |
|  | | Syphilitic Myocarditis | 093.82 |  |
|  | | Myocarditis due to Toxoplasmosis | 130.3 |  |
|  | | Acute Rheumatic Myocarditis | 391.2 |  |
|  | | Rheumatic Myocarditis | 398.0 |  |
|  | | Acute Myocarditis | 422.0 |  |
|  | | Acute Myocarditis, Unspecified | 422.90 |  |
|  | | Idiopathic Myocarditis | 422.91 |  |
|  | | Septic Myocarditis | 422.92 |  |
|  | | Toxic Myocarditis | 422.93 |  |
|  | | Myocarditis, Unspecified | 429.0 |  |
|  | | Dilated Cardiomyopathy/Other Primary Cardiomyopathies | 425.4 |  |
|  | | Hypertrophic Cardiomyopathy | 425.1 |  |
|  | | Nutritional/Metabolic Cardiomyopathies | 425.7 |  |
|  | | Cardiomyopathy in Other Diseases Classified Elsewhere | 425.8 |  |
| PH | | Primary Pulmonary Arterial Hypertension | 416.0 |  |
|  | | Other Chronic Pulmonary Heart Diseases | 416.8 |  |
|  | | Other Specified Diseases of Pulmonary Circulation | 417.8 |  |

*BV = biventricular, CHD = congenital heart disease, CM = myocarditis/cardiomyopathy, PH = pulmonary hypertension, SV = single ventricle.*

| Supplemental Table 2. Models of Daily Bleeding, Controlling for Site and Cardiac Diagnosis | | | | |
| --- | --- | --- | --- | --- |
|  | | **Bleeding event** | | |
|  | Overall (N = 1088) | Odds ratio (95% CI) | P-value | % Missing |
| **Age Group** |  |  | 0.095 | 0% |
| pre-term neonate | 84 (7.7%) | 1.42 (0.77, 2.61) |  |  |
| full-term neonate | 380 (34.9%) | Reference |  |  |
| infant | 311 (28.6%) | 0.69 (0.40, 1.21) |  |  |
| child | 214 (19.7%) | 0.96 (0.45, 2.07) |  |  |
| adolescent | 99 (9.1%) | 0.30 (0.10, 0.95) |  |  |
| **E-CPR** | 284 (26.1%) | 1.34 (0.74, 2.43) | 0.331 | 0% |
| **Type of Pump** |  |  | 0.857 | 0% |
| Roller Head | 294 (27.0%) | 1.09 (0.42, 2.83) |  |  |
| Centrifugal | 794 (73.0%) | Reference |  |  |
| **ECMO Flow Rate (20 mL/kg/min)** | 103.7 [82.5, 129.6] | 1.06 (0.99, 1.14) | 0.096 | 1% |
| **Central Cannulation** | 489 (44.9%) | 1.83 (1.03, 3.24) | 0.039 | 0% |
| **Mediastinal Exploration During ECMO** | 32 (2.9%) | 1.02 (0.41, 2.55) | 0.968 | 0% |
| **STAT Category > 2** | 495 (45.5%) | 1.21 (0.69, 2.15) | 0.505 | 0% |
| **Heparin Dose (10 IU/kg/hour)** | 23.5 [14.2, 33.0] | 0.95 (0.83, 1.09) | 0.464 | 0% |
| **Cannulation from CPB** |  |  | 0.010 | 0% |
| Day after cannulation, from CPB | 48 (4.4%) | 2.25 (1.27, 3.98) |  |  |
| Day after cannulation, not from CPB | 139 (12.8%) | 1.31 (0.91, 1.90) |  |  |
| > 1 day after cannulation | 901 (82.8%) | Reference |  |  |
| **Blood Urea Nitrogen (10 mg/dL)** | 22.0 [13.0, 37.0] | 0.96 (0.87, 1.06) | 0.448 | 2% |
| **Creatinine (mg/dL)** | 0.6 [0.4, 0.9] | 1.11 (0.78, 1.59) | 0.545 | 2% |
| **Aspartate Aminotransferase (100 IU/L)** | 126.5 [76.0, 299.0] | 1.01 (1.00, 1.02) | 0.130 | 30% |
| **Alanine Aminotransferase (100 IU/L)** | 41.5 [26.5, 90.0] | 1.01 (0.97, 1.04) | 0.649 | 29% |
| **Lactate (mmol/L)** | 1.9 [1.3, 4.2] | 1.04 (1.01, 1.07) | 0.006 | 8% |
| **ACT (50 seconds)** | 201.0 [186.0, 223.0] | 1.09 (1.03, 1.16) | 0.005 | 2% |
| **aPTT (10 seconds)** | 76.4 [55.4, 120.0] | 1.01 (0.98, 1.05) | 0.486 | 20% |
| **PT (seconds)** | 15.6 [13.9, 18.2] | 1.04 (1.01, 1.07) | 0.002 | 23% |
| **INR (0.5)** | 1.3 [1.2, 1.6] | 1.22 (1.08, 1.37) | 0.001 | 23% |
| **Anti-Xa (0.3 IU/mL)** | 0.3 [0.1, 0.4] | 1.09 (0.85, 1.39) | 0.507 | 53% |
| **Fibrinogen (100 mg/dL)** | 225.0 [157.0, 310.5] | 0.70 (0.60, 0.82) | <.001 | 7% |
| **Platelets (10 x 10^3^/µL)** | 95.0 [68.5, 123.0] | 0.97 (0.93, 1.00) | 0.038 | 1% |
| **ACT Compared to Goal** |  |  | 0.008 | 0% |
| Low | 149 (13.7%) | 0.88 (0.58, 1.35) |  |  |
| Target | 400 (36.8%) | Reference |  |  |
| High | 288 (26.5%) | 1.48 (1.03, 2.12) |  |  |
| Not assessed, > 1 day after cannulation | 64 (5.9%) | 1.28 (0.60, 2.71) |  |  |
| Not assessed, day after cannulation | 187 (17.2%) | 1.76 (1.21, 2.58) |  |  |
| **Platelet Count Compared to Goal** |  |  | 0.021 | 2% |
| Low | 312 (29.1%) | 0.99 (0.74, 1.32) |  |  |
| Target | 572 (53.4%) | Reference |  |  |
| Not assessed, day after cannulation | 187 (17.5%) | 1.58 (1.13, 2.20) |  |  |

*ACT = activated clotting time, aPTT = activated partial thromboplastin time, CPB = cardiopulmonary bypass, ECMO = extracorporal membrane oxygenation, E-CPR = extracorporeal cardiopulmonary resuscitation, INR = international normalized ratio, PT = prothrombin time, STAT = Society of Thoracic Surgeons-European Association of Cardiothoracic Surgery.*
